# Changes in plasma free fatty acids in obese patients before and after bariatric surgery highlight alterations in lipid metabolism

**DOI:** 10.1038/s41598-022-19657-9

**Published:** 2022-09-12

**Authors:** Stephen J. Hierons, Kazim Abbas, Amélie I. S. Sobczak, Michela Cerone, Terry K. Smith, Ramzi A. Ajjan, Alan J. Stewart

**Affiliations:** 1 School of Medicine, University of St Andrews, St Andrews, KY16 9TF UK; 2 Renal Transplant Unit, Manchester Royal Infirmary, Manchester, UK; 3 Biomedical Sciences Research Complex, University of St Andrews, St Andrews, KY16 9ST UK; 4 Leeds Institute of Cardiovascular and Metabolic Medicine, University of Leeds, Leeds, UK

**Keywords:** Lipidomics, Lipids, Obesity, Outcomes research, Molecular medicine

## Abstract

Obesity is a complex disease that increases an individual’s risk of developing other diseases and health-related problems. A common feature is dyslipidemia characterized by increased levels of plasma lipids, which include non-esterified fatty acids (NEFAs). The role of NEFAs in obesity-related morbidity is interesting as NEFAs constitute a reservoir of metabolic energy, are principal components of cell membranes and are precursors for signalling molecules. Bariatric surgery promotes sustained weight loss in severely obese patients, reducing the incidence and severity of co-morbidities. In this study we measure changes in circulating NEFA species in plasma samples taken from 25 obese individuals before and 9 months after Roux-en-Y gastric bypass surgery. The mean weight of the cohort reduced by 29.2% from 149.0 ± 25.1 kg pre-surgery to 105.5 ± 19.8 kg post-surgery and the BMI by 28.2% from 51.8 ± 6.3 kg/m^2^ pre-surgery to 37.2 ± 5.4 kg/m^2^. Mean glycated haemoglobin (HbA1c) reduced from 6.5 ± 1.3 to 5.5 ± 0.5%, consistent with the intervention leading to improved glycaemic control, particularly in those who were dysglycemic prior to surgery. Total and LDL cholesterol concentrations were markedly reduced following surgery. Concentrations of seven NEFAs were found to decrease 9 months after surgery compared to pre-surgery levels: myristate, palmitoleate, palmitate, linoleate, oleate, stearate and arachidonate. Bariatric surgery led to increased lipogenesis and elongase activity and decreased stearoyl-CoA desaturase 1 activity. This study therefore highlights metabolic changes that take place following gastric bypass surgery in severely obese patients.

## Introduction

Obesity is a complex disease associated with excessive body fat that increases an individual’s risk of developing other diseases and health-related problems^1^. These include heart disease^2^, type-2 diabetes^3,4^, thrombotic disorders including stroke^5^, high blood pressure^6^ and certain cancers^7,8^. A common feature of obesity is dyslipidemia, characterized by increased triglycerides, triglyceride-rich lipoproteins, and HDL cholesterol^9^. The role of non-esterified fatty acids (NEFAs; also often referred to as free fatty acids) in obesity-related morbidity is also of interest. Circulating NEFAs, which are carried in plasma by serum albumin^10^, act as a major reservoir of metabolic energy, are a principal component of cell membranes and are precursors for signalling molecules^11^. Obesity is known to lead to an increase in plasma NEFA concentrations^12^, which has been linked to adverse metabolic outcomes^13,14^. Thus, changes in the composition of plasma NEFAs can potentially cause further complications in individuals with obesity and reflect a wider dysregulation of lipid metabolism.

Roux-en-Y gastric bypass surgery has been shown to promote sustained weight loss in severely obese patients, reduce the incidence and severity of co-morbidities^15^ and increase life expectancy^16^. Indeed, the surgery is associated with rapid remission of type II diabetes (often occurring just days after surgery)^17^. Roux-en-Y is a laparoscopic surgery that reduces the size of the stomach by ~ 80%^18^; its metabolic benefits occur through a combination of reduced dietary intake and malabsorption^19^. Benefits may also incur from surgery-induced adaptations in gastrointestinal physiology. This includes changes to the gut microbiome and alterations in gut and pancreatic peptide hormone levels^20^. The surgery has been shown to reduce plasma triglycerides, cholesterol, and cholesterol LDL levels, thus reversing the dyslipidemic picture commonly observed in the obese^21^. The effect of Roux-en-Y surgery on plasma NEFA composition however has been less comprehensively studied.

In this study, we measure changes in circulating NEFA species in plasma samples taken from obese patients before and 9 months after Roux-en-Y bariatric surgery. This represents the time post-surgery when excess weight loss is expected to plateau^22^. Concentrations of individual NEFA species have also been used to calculate indices reflecting desaturation, elongation and de novo lipogenesis activity within adipocytes. We explore how plasma NEFA concentrations and lipid indices are influenced by the surgical intervention and reflect on how the resultant changes relate to the metabolic modifications that occur.

## Results and discussion

### Demographic and anthropometric information of study participants before and 9 months after bariatric surgery

Anthropometric values (including weight, BMI, waist/hip/neck circumferences and waist-to-hip ratio, visceral fat rating and excess weight) and whole-blood concentrations of HbA1c were measured and compared in the 25 participants before and 9 months after Roux-en-Y surgery. Table 1 summarizes the demographic characteristics of the cohort as well as anthropometric values before and after surgery.

**Table 1:** Demographic characteristics of the studied participants (n = 25) and mean anthropometric values before and 9 months after surgery.

Characteristics	Values			
Age at recruitment (years ± SD)	46.2 ± 10.3			
% males (number)	36.0 (9 out of 25)			
% smokers (number)	12.0 (3 out of 25)			

	Pre-surgery	9 months post-surgery	p-value	Significance
Weight (kg ± SD)	149.0 ± 25.1	105.5 ± 19.8	< 0.0001	****
BMI (kg/m^2^ ± SD)	51.8 ± 6.3	37.2 ± 5.4	< 0.0001	****
Waist circumference (cm ± SD)	143.7 ± 11.2	117.7 ± 12.9	< 0.0001	****
Hip circumference (cm ± SD)	135.9 ± 11.3	116.8 ± 10.8	< 0.0001	****
Waist-to-hip (ratio ± SD)	1.07 ± 0.08	1.01 ± 0.08	0.0004	***
Neck circumference (cm ± SD)	45.8 ± 5.1	40.2 ± 4.5	< 0.0001	****
Visceral fat rating (rating ± SD)	22.1 ± 7.1	13.2 ± 4.1	< 0.0001	****
Excess weight (kg ± SD)	87.0 ± 22.1	44.1 ± 16.7	< 0.0001	****

The p-values were calculated using paired T-test. Statistical significance is indicated as: ***p ≤ 0.001 and ****p ≤ 0.0001.

In the 9 months following surgery, the mean weight of the cohort reduced by 29.2% from 149.0 ± 25.1 kg pre-surgery to 105.5 ± 19.8 kg post-surgery (p < 0.0001) and the BMI by 28.2% from 51.8 ± 6.3 kg/m^2^ pre-surgery to 37.2 ± 5.4 kg/m^2^ (p < 0.0001). Significant differences were also observed in the waist, hip and neck circumference, with the waist-to-hip ratio decreasing from 1.07 ± 0.08 to 1.01 ± 0.08 (p < 0.0001). The visceral fat rating decreased from 22.1 ± 7.1 to 13.2 ± 4.1 (p < 0.0001) 9 months after surgery; note that a rating between 1 and 12 indicates a healthy level of visceral fat, whereas a rating between 13 and 59 indicates excess visceral fat. Excess fat was reduced by 49.3% from 87.0 ± 22.1 kg pre-surgery to 44.1 ± 16.7 kg (p < 0.0001) 9 months after surgery. These measurements indicate the treatment was successful in aiding substantive weight loss by 9 months post-surgery.

### Glycated haemoglobin (HbA1c), total cholesterol, triglyceride, HDL and LDL concentrations before and 9 months after bariatric surgery

**Figure 1:**
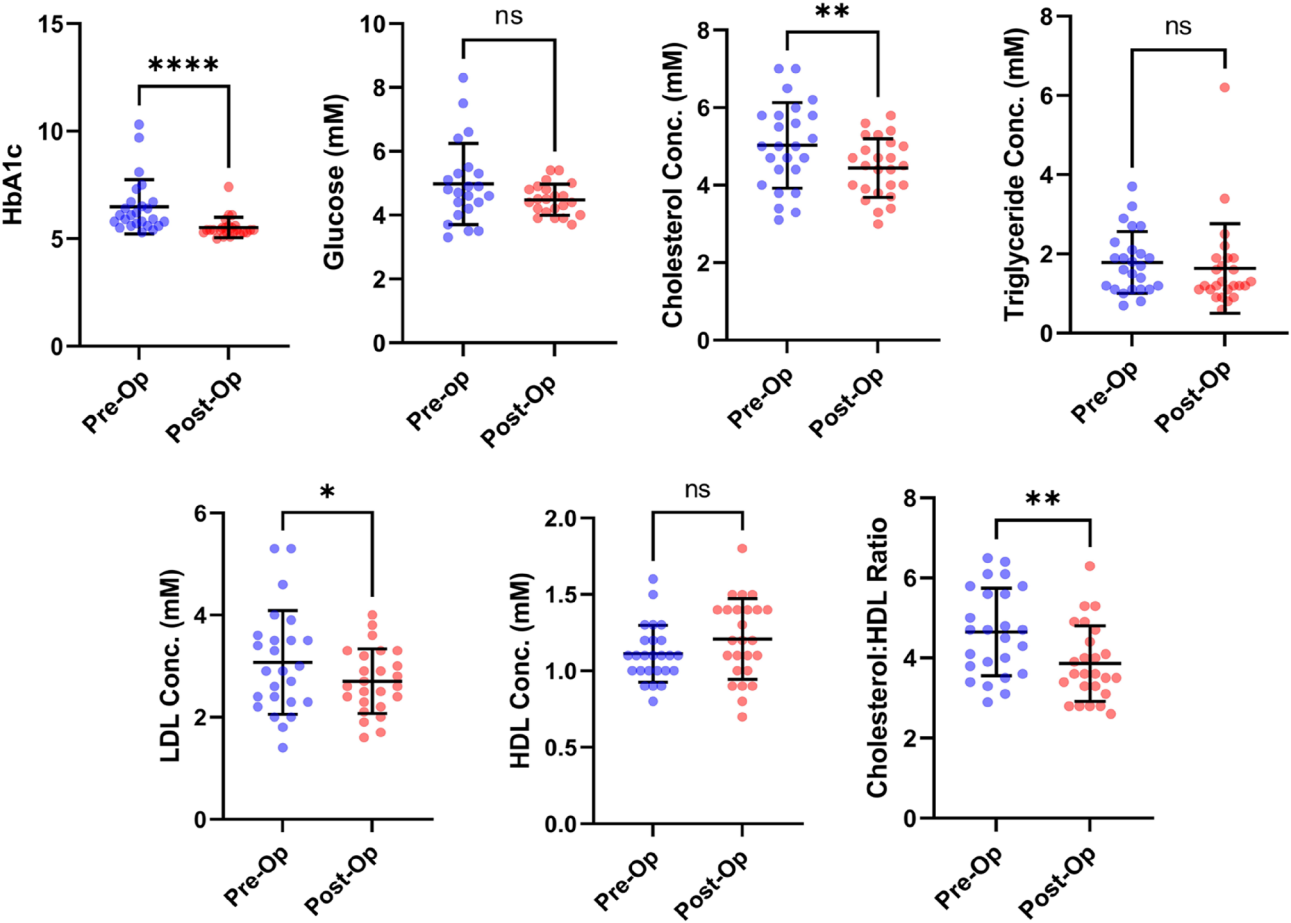
Mean plasma HbA1c, total cholesterol, triglyceride, HDL and LDL concentrations in participants before (Pre-Op) and 9 months after (Post-Op) bariatric surgery. Error bars show the means values ± standard deviation. The p-values were calculated using paired T-test. Statistical significance is indicated as: *ns* not significant (p > 0.05), *p ≤ 0.05 and **p ≤ 0.01, ***p ≤ 0.001 and ****p ≤ 0.0001.

Plasma HbA1c, glucose, total cholesterol, triglyceride, HDL and LDL cholesterol concentrations were measured in the participants before and 9 months after Roux-en-Y surgery (Fig. 1; Table 2). HbA1c concentrations for most of the participants were previously reported as part of another study focused on metal micronutrient status^23^. Whole-blood HbA1c for the 25 participants in the study presented here was reduced from 6.5 ± 1.3% to 5.5 ± 0.5% (p < 0.0001), consistent with the intervention leading to improved glycaemic control in the cohort. Indeed, 13 of the participants (52%) were dysglycemic (defined as an HbA1c concentration ≥ 6.0%) prior to surgery, while only 3 (12%) remained dysglycemic 9 months after surgery. A greater reduction in HbA1c concentration was observed in those with dysglycemia before surgery. Prior to surgery, 7 of the participants were prescribed hypoglycemic agents, while at 9-months post-surgery, only 2 required these therapies. This is reflective of the normalized glycaemic status in some of the participants and is consistent with other studies that have also reported that bariatric surgery, and the substantive weight loss that results from it, aids in reducing elevated HbA1c levels to normal levels^24,25^. Glucose concentrations were not significantly altered after surgery.

**Table 2:** Mean HbA1c, glucose total cholesterol, triglyceride, HDL and LDL concentrations and prescribed medications in participants before and 9 months after bariatric surgery.

	Pre-surgery	9 months post-surgery	p-value	Significance
HbA1c (% ± SD)	6.5. ± 1.3	5.5 ± 0.5	< 0.0001	****
Glucose (mmol/l ± SD)^a^	4.977	4.482	0.0727	ns
% with dysglycemia (number)	52% (13/25)	12% (3/25)	N/A	N/A
% prescribed metformin (number)	28% (7/25)	8% (2/25)	N/A	N/A
Total cholesterol (mmol/l ± SD)	5.0 ± 1.1	4.4 ± 0.8	0.0059	**
Triglyceride (mmol/l ± SD)	1.8 ± 0.8	1.6 ± 1.1	0.5331	ns
HDL (mmol/l ± SD)	1.1 ± 0.2	1.2 ± 0.3	0.0575	ns
LDL (mmol/l ± SD)	3.1 ± 1.0	2.7 ± 0.6	0.0285	*
Cholesterol:HDL ratio (ratio ± SD)	4.7 ± 1.1	3.9 ± 0.9	0.0026	**
% prescribed statin (number)	28% (7/25)	24% (6/25)	N/A	N/A

The p-values were calculated using paired T-test. Statistical significance is indicated as: *ns* not significant (p > 0.05), *p ≤ 0.05 and **p ≤ 0.01, ***p ≤ 0.001 and ****p ≤ 0.0001. N/A for not applicable. ^a^Measurements of blood glucose conc. before and after surgery were only available in 22 of the 25 individuals.

Total cholesterol concentration in the cohort decreased from 5.0 ± 1.1 mmol/l pre-surgery to 4.4 ± 0.8 mmol/l 9 months after surgery (p = 0.0059). Mean LDL and total cholesterol:HDL ratio also decreased after bariatric surgery. LDL concentration was 3.1 ± 1.0 mmol/l pre-surgery and 2.7 ± 0.6 mmol/l 9 months after surgery (p = 0.0285). The cholesterol:HDL ratio was 4.7 ± 1.1 mmol/l pre-surgery and 3.9 ± 0.9 mmol/l 9 months after surgery (p = 0.0026). Mean triglyceride and HDL concentrations were unchanged. Although mean concentrations of total cholesterol, triglyceride, HDL and LDL cholesterol were in the healthy range before and after surgery, eleven participants had total cholesterol levels greater than 5 mmol/l (deemed an unhealthy concentration) before surgery but only 6 had higher levels than this 9 months after surgery. Prior to surgery, 7 were prescribed a statin and 6 were still taking one at 9-months post-surgery a statin. The surgical intervention (together with taking a statin in those that were prescribed these drugs) led to reductions in total cholesterol and LDL levels, with the decrease in the former also reflected in the reduced total cholesterol:HDL ratio. Reduction of total cholesterol and LDL level are each well known to be associated with a decrease in the risk of adverse cardiovascular events^26^. Overall, the data suggests a fundamental improvement in lipid metabolism in our clinical cohort, a finding which is consistent with previous studies^27^.

### Plasma non-esterified fatty acid concentrations in participants before and 9 months after bariatric surgery

**Figure 2:**
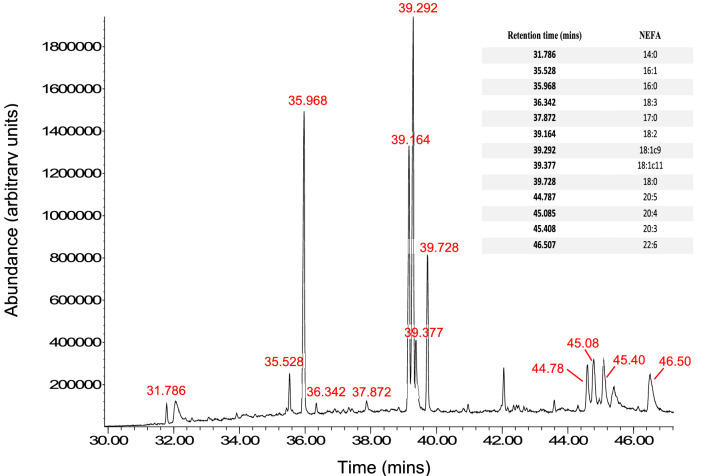
Example GC:MS chromatogram showing points of separation of individual FAMEs. The retention times of peaks corresponding to individual NEFA species are shown in the table.

**Figure 3:**
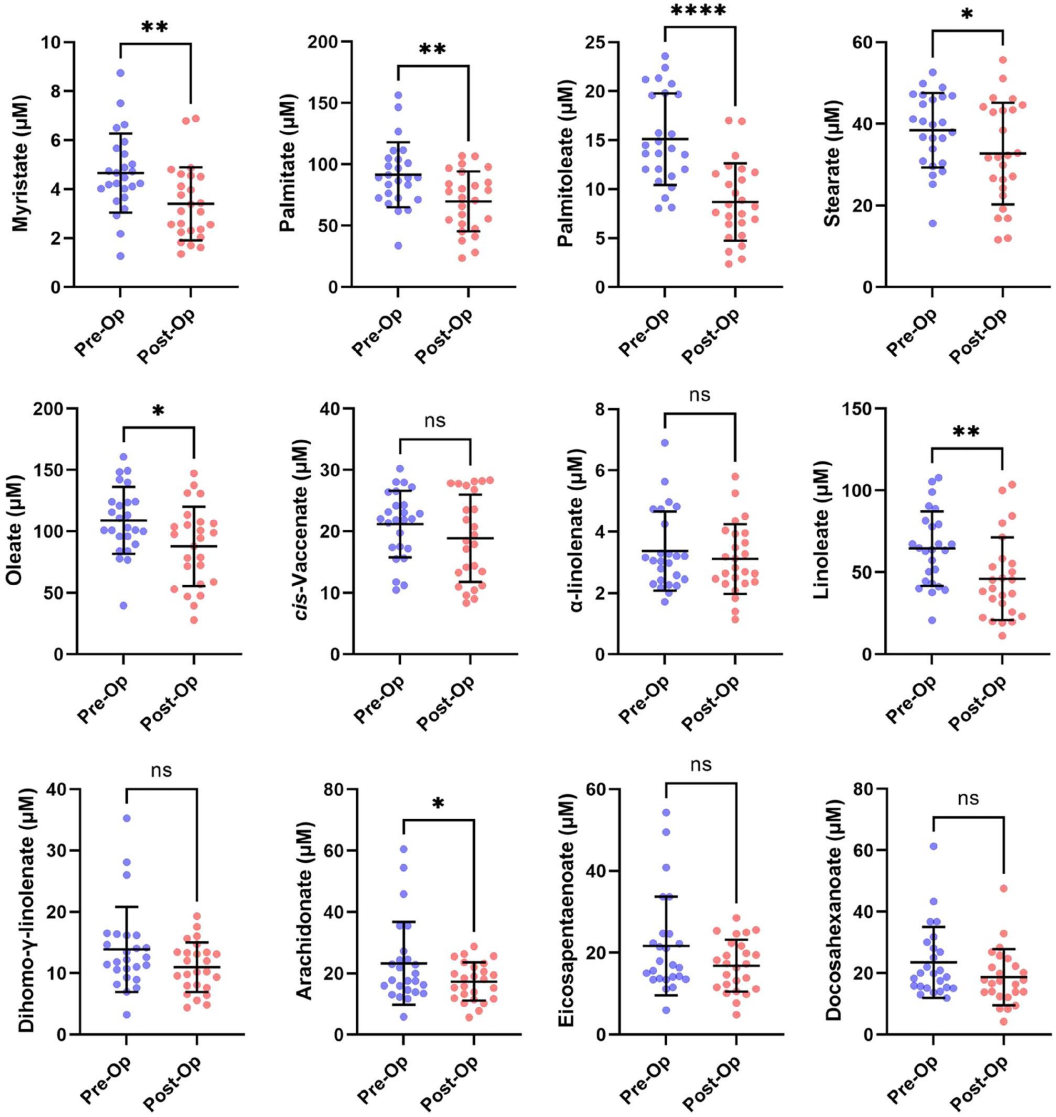
Mean plasma NEFA concentrations in participants before (Pre-Op) and 9 months after (Post-Op) bariatric surgery. Error bars show the mean values ± standard deviation. The p-values were calculated using paired T-test. Statistical significance is indicated as: *ns* not significant (p > 0.05), *p ≤ 0.05 and **p ≤ 0.01, ***p ≤ 0.001 and ****p ≤ 0.0001.

**Figure 4:**
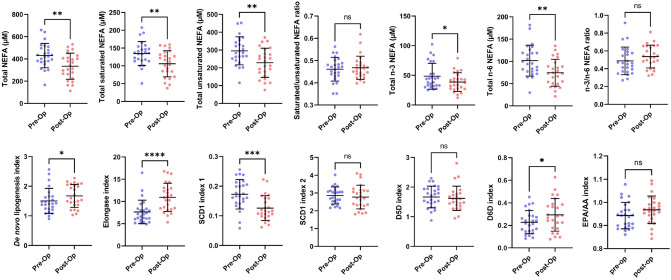
Mean plasma NEFA indices and ratios in participants before (Pre-Op) and 9 months after (Post-Op) bariatric surgery. Error bars show the mean values ± standard deviation. The p-values were calculated using paired T-test. Statistical significance is indicated as: ns, not significant (p > 0.05), *p ≤ 0.05 and **p ≤ 0.01, ***p ≤ 0.001 and ****p ≤ 0.0001.

Following the conversion of plasma NEFAs to fatty acid methyl esters (FAMEs), NEFA concentrations in plasma samples taken from participants before and 9 months after Roux-en-Y surgery were measured. An example GC-MS chromatogram showing the retention times of individual NEFA peaks is shown in Fig. 2. The resultant quantitative data for the respective NEFA species in each of the samples is presented (Fig. 3; Table 3). The concentrations of seven NEFAs were found to decrease 9 months after surgery compared to pre-surgery levels: myristate (14:0; p < 0.0019), palmitoleate (16:1; p < 0.0001), palmitate (16:0; p < 0.0033), linoleate (18:2; p < 0.0013), oleate (18:1c9; p < 0.0110), stearate (18:0; p < 0.0420) and arachidonate (20:4; p = 0.0345). The other five major NEFA species measured exhibited no difference in concentration 9 months after surgery compared to pre-surgery levels. These were α-linolenate (18:3), cis-vaccenate (18:1c11), eicosapentaenoate (20:5), dihomo-γ-linoleate (20:3) and docosahexaenoate (22:6). Further analysis of the data revealed a reduction in concentrations of total NEFAs (p < 0.0019), total saturated NEFA (p < 0.0028), total unsaturated NEFA (p < 0.0022), total n-3 NEFA (p < 0.0406) and total n-6 NEFA (p < 0.0022) at 9 months after surgery compared to pre-surgery concentrations (Fig. 4). The total saturated/unsaturated and n-3/n-6 NEFA ratios were unchanged.

**Table 3:** Mean plasma NEFA concentrations and indices in participants before and 9 months after bariatric surgery.

	Pre-surgery	9 months post-surgery	p-value	Significance
**Non-esterified fatty acids (μmol/l ± SD)**				
Myristate, 14:0	4.7 ± 1.6	3.4 ± 1.5	0.0019	**
Palmitate, 16:0	91.6 ± 26.6	69.8 ± 24.5	0.0033	**
Palmitoleate, 16:1	15.1 ± 4.7	8.7 ± 3.9	< 0.0001	****
Stearate, 18:0	38.5 ± 9.1	32.7 ± 12.5	0.0420	*
Oleate, 18:1c9	108.9 ± 27.3	87.8 ± 32.3	0.0110	*
*cis*-Vaccenate, 18:1c11	21.2 ± 5.4	18.9 ± 7.1	0.1994	ns
Linoleate, 18:2 (n-6)	64.5 ± 22.7	46.1 ± 25.2	0.0013	**
α-Linolenate, 18:3 (n-3)	3.4 ± 1.3	3.1 ± 1.1	0.4018	ns
Dihomo-γ-linolenate, 20:3 (n-6)	23.5 ± 11.5	18.7 ± 9.1	0.0633	ns
Arachidonate, 20:4 (n-6)	23.3 ± 13.5	17.3 ± 6.2	0.0345	*
Eicosopentanoate, 20:5 (n-3)	21.7 ± 12.1	16.8 ± 6.3	0.0616	ns
Docohexanoate, 22:6 (n-3)	13.9 ± 6.9	11.0 ± 4.0	0.0652	ns
**NEFA classes (μmol/l ± SD)**				
Total NEFA	430.1 ± 109.7	334.4 ± 117.2	0.0019	**
Total saturated NEFA	134.7 ± 33.7	105.9 ± 36.7	0.0028	**
Total unsaturated NEFA	295.4 ± 78.4	228.4 ± 81.8	0.0022	**
Total n-3 NEFA	48.5 ± 21.8	38.6 ± 15.7	0.0406	*
Total n-6 NEFA	101.7 ± 34.8	74.4 ± 30.2	0.0022	**
**NEFA ratios and indices (ratio ± SD)**				
Saturated/unsaturated NEFA ratio	0.46 ± 0.05	0.47 ± 0.05	0.6541	ns
n-3/n-6 NEFA ratio	0.49 ± 0.16	0.54 ± 0.13	0.1584	ns
EPA/AA ratio (20:5/20:4)	0.94 ± 0.06	0.97 ± 0.06	0.1913	ns
De novo lipogenesis index (16:0/18:2)	1.50 ± 0.42	1.67 ± 0.39	0.0360	*
Elongase index (18:1c9/16:1)	7.64 ± 2.65	10.92 ± 3.17	< 0.0001	****
SCD1 index 1 (16:1/16:0)	0.17 ± 0.05	0.13 ± 0.04	0.0001	***
SCD1 index 2 (18:1c9/18:0)	2.86 ± 0.48	2.77 ± 0.67	0.6168	ns
D5D index (20:4/20:3)	1.67 ± 0.36	1.62 ± 0.41	0.6990	ns

The p-values were calculated using paired T-test. Statistical significance is indicated as: *ns* not significant (p > 0.05), *p ≤ 0.05 and **p ≤ 0.01, ***p ≤ 0.001 and ****p ≤ 0.0001.

Different plasma NEFA indices were then calculated and levels before and after surgery were compared. The de novo lipogenesis index was calculated as the ratio of palmitate (16:0, the main product of lipogenesis) with linoleate (18:2, an essential NEFA only found in the diet). The elongase index, which indicates elongase enzyme activity was calculated as the oleate/palmitoleate (18:1c9/16:1) ratio. The activity of stearoyl-CoA desaturase 1 (SCD1) was calculated with the SCD1 index 1 and 2, which were the palmitoleate/palmitate (16:1/16:0) ratio and the oleate/stearate (18:1c9/18:0) ratio respectively. The delta-(5)-desaturase (D5D) and delta-(6)-desaturase (D6D) indices were calculated with the arachidonate/dihomo-γ-linolenate (20:4/20:3) ratio and the α-linolenate/linoleate (18:3/18:2) ratio respectively. The ratio of eicosapentaenoate to arachidonate (EPA/AA ratio), a marker of cardioprotective health was also measured. An increase in the de novo lipogenesis (p = 0.0360), elongase (< 0.0001) and D6D (p = 0.0382) indices and a decrease in the SCD1 index 1 (p = 0.0001) were observed following surgery. No change was observed in the SCD1 index 2, D5D index or EPA/AA ratio at 9 months after surgery compared to pre-surgery levels.

Several other studies that have examined plasma NEFA profiles in patients after various types of bariatric surgery have found such interventions to lead to significant reductions in total NEFA concentrations (and groups of fatty acids) in the months following surgery^28–33^, in a manner similar to that observed in this study. A meta-analysis published in 2016 found that serum NEFA levels were decreased at 6 and 12 months but did not differ from preoperative levels at 3 months post-surgery^34^. However, this analysis also found that serum NEFAs did not differ significantly from pre-operative levels at 18 and 24 months after surgery. This work (and the data presented here) suggests that the greatest reduction in circulatory NEFA concentration is observed between 6 and 12 months after bariatric surgery. Reductions in plasma NEFA concentrations post-surgery likely occur due to greater sensitivity of the adipocyte to the anti-lipolytic effects of insulin (which although not directly measured, would be expected given the observed reductions in % glycated haemoglobin levels). It is worth noting that Roux-en-Y surgery is also known to alter the gut microbiota^35^, which has the potential to influence circulating NEFA levels.

The effect of Roux-en-Y surgery on NEFA composition in the plasma has not been extensively studied. Of particular interest is the effect of the surgery on the two essential fatty acids, linoleate (18:2n-6, an omega-6 fatty acid) and α-linolenate (18:3n-3, an omega-3 fatty acid), and also their polyunsaturated fatty acid derivatives. Specifically, linoleate is converted to arachidonic acid (20:4n-6) and α-linolenate is converted to eicosapentaenoic (20:5n-3) acid and docosahexaenoic acid (22:6n-3). All four NEFAs were measured in our study. Of these, linoleate was found to be present in plasma at reduced levels at 9 months following surgery. This NEFA has been shown to be associated with a lower incidence of type 2 diabetes^36^, and coronary heart disease mortality^37^. This observed decrease in linoleate concentration suggests that the reduction in the effective size of the stomach may lead to reduced absorption of this essential fatty acid. The concentration of arachidonate was also reduced at 9 months after surgery. Arachidonate has essential roles in the brain and serves as a precursor for eicosanoid biosynthesis^38^. Our study found no significant difference in eicosapentaenoic acid and docosahexaenoic acid between pre and post-surgery. Our study however does report a reduction in both total omega-3 and omega-6 fatty acids. A recent systematic review has analyzed the effect of different obesity surgeries on blood polyunsaturated NEFAs. There are some conflicting data in the current literature (both increases and decreases in polyunsaturated NEFAs have been reported following obesity surgery^39^). Our own analysis of the current literature confirms this (Table 4). Overall, however, the authors concluded that both the essential fatty acids and eicosapentaenoic acid decrease in the months following surgery. Interestingly, the effect of Roux-en-Y on levels of saturated NEFAs also appears to vary depending on the study. Differences in the observed fatty acid profile following surgery are likely due to several factors including but limited to; the type of surgical procedure (and hence the degree of surgery-induced restriction/malabsorption and/or alteration to gastrointestinal physiology, the clinical characteristics of the studied population (including the presence of relevant comorbidities that may affect lipid metabolism), the method of NEFA quantification, the follow up period and differences in diet/supplementation following surgery.

**Table 4:** Summary of previous studies investigating the effect of Roux-en-Y surgical intervention on plasma/serum NEFA composition in obese patients at various time points.

Study	Number of participants	Pool in which NEFA was measured	Measured NEFA units	Time point measured	Effect of Roux-en-Y Surgery on NEFA composition
Mutch et al.^40^	14	Serum	Arbitrary units based on metabolomics	Pre-surgery, 3 months and 6 months post-surgery	Saturated NEFAs (14:0, 18:0, 22:0 and 23:0) were significantly lower at 3 and 6 months compared to the pre-surgery period. Levels of γ-linolenic acid were also lower at 3 and 6 months following Roux-en-Y. Most unsaturated NEFAs (18:1c9 trans, 20:4n-6, 22:6n-3 and 24:1n-9) were significantly higher at 3 and 6 months compared to the pre-operative period. Individual NEFA levels did not significantly differ between 3 and 6 months.
Lopes et al.^41^	10	Plasma	N/A	Pre-surgery and 12 months post-surgery	Saturated NEFAs (14:0, 15:0, 16:0, 17:0, 18:0, and 22:0) were significantly higher at 12 months post-surgery. Polyunsaturated NEFAs (18:3n-3, 18:2n-6, 20:5n-3, 20:4n-3, 20:3n-6, and 22:6n-3) were significantly lower 12 months post-surgery.
Hovland et al.^42^	34	Plasma	%Weight	Pre-Surgery and 12 months post-surgery	Saturated NEFAs (14:0, 16:0 and 18:0) were significantly lower at 12 months post-surgery. Several monounsaturated NEFAs (16:1n-7 and 16:1n-9) decreased following surgery while others (18:1n-7 and 24:1n-9) increased Omega-3 polyunsaturated NEFAs (22:5n-3 and 22:6n-3) and omega-6 polyunsaturated NEFAs (18:3n-6, 20:3n-6 and 22:5n-6) increased after surgery.
Sarkar et al.^43^	21	Serum	Absolute units (ug/ml)	Pre-surgery, 6 months and 18 months post-surgery	All saturated NEFAs (14:0, 16:0 and 18:0) and most measured monounsaturated NEFAs (16:1, 18:1n-7, and 18:1n-9) and polyunsaturated NEFAs (18:2n-6, 20:3n-6 and 20:4n-6) decreased at 6- and 18-months post-surgery.
Wijayatunga et al.^44^	8	Serum	Absolute units (mg/ml)	Pre-surgery and 6 months post-surgery	Saturated NEFAs (10:0, 13:0, 14:0, 15:0, and 18:0) were significantly increased at 6-months post-surgery. The study did not detect significant changes in essential NEFAs or omega-3 polyunsaturated NEFAs.
Thomas et al.^45^	11	Plasma	Reported as % change	Pre-surgery and 3 days post-surgery	Of the 6 NEFAs measured (palmitoleic, palmitic, margaric, linoleic, oleic and stearic acid,), there was a significant decrease in palmitic (16:0) and linoleic acid (18:2n-6) at 3-days post-surgery.

To fully understand changes in lipid metabolism following Roux-en-Y surgery, different fatty acid indices were calculated to indicate the activities of various enzyme classes involved in fatty acid metabolism. The increase in de novo lipogenesis index at 9 months following surgery might suggest an increase in fatty acid synthesis. This is supported by a study that found that bariatric weight loss increased de novo lipogenesis in white adipose tissue^46^. However, it is more likely this result is due to lower consumption and reduced absorption of dietary fat after surgery given that palmitate concentrations (the major product of de novo lipogenesis) did not increase post-surgery and the essential linoleic acid (found only in the diet) was lower following surgery. The elongase index and the D6D index increased following surgery. The D5D index was unchanged between the groups. The increased elongase activity is consistent with reports demonstrating that weight loss, induced by bariatric surgery, leads to increased expression of various genes involved in fatty acid metabolism in adipose tissue including fatty acid elongase-6 and fatty acid synthase^47^. The D6D index can reflect an increased production of polyunsaturated fatty acids. However, in our case, this parameter most likely increased because of a much-reduced level of plasma linoleate after surgery due to restricted dietary intake. The fact that the D5D index did not change (which can also reflect polyunsaturated fatty acid synthesis) supports this. The SCD1 index 1 was found to be significantly decreased after surgery. However, SCD1 index 2 was unchanged. These indices are reflective of stearoyl-CoA desaturase 1 activity, an enzyme known to exhibit increased expression in obesity^48^. This enzyme has been shown to exhibit decreased activity following weight loss, but the decrease in activity (at least regarding palmitoleate to palmitate conversion) can also potentially be attributable to decreased carbohydrate intake^49^.

## Conclusions

In summary, we show that Roux-en-Y gastric surgery leads to substantive weight loss and corresponding changes in anthropometric parameters in obese patients 9 months following surgery. The intervention additionally led to the normalization of dysglycemia in several participants. The total and LDL cholesterol concentrations were markedly reduced following surgery. The study identified reductions in the plasma concentrations of most of the NEFA species examined, including saturated, monounsaturated and polyunsaturated species. Measurement of individual NEFAs revealed specific changes in lipid metabolism 9 months after surgery that included increased lipogenesis in addition to increased elongase and decreased stearoyl-CoA desaturase 1 activity. The novel findings presented here further illuminate the metabolic changes that take place following gastric bypass surgery in severely obese patients.

## Materials and methods

### Ethics statement

Blood samples were collected following approval by the National Research Ethics Service Committee Yorkshire & The Humber—Sheffield (REC reference: 11/H1308/16) after obtaining written informed consent from all participants. All research protocols were performed in accordance with the Declaration of Helsinki.

### Sample collection and treatment

Briefly, a total of 25 Roux-en-Y surgical patients (17 females and 8 males) were recruited from York Hospital, York, United Kingdom. Male or female participants over 18 years of age and referred to the hospital for bariatric surgery for obesity were included in this study. Exclusion criteria included if participants were under 18 years old, were diagnosed with an endocrine disorder other than type 2 diabetes, had a history of alcohol or drug abuse, had a significant psychological history, had a history of deep vein thrombotic disease, were taking warfarin, were pregnant, had a history of active malignancy or if they developed post-operative complications. Two weeks before surgery the patients were placed on a low-calorie diet (800–1000 kcal/day). At this point, they also commenced taking multi-vitamin/mineral supplements. After surgery patients were given a puree diet for 4 weeks and after this time, advised to continue taking multi-vitamin/mineral supplement. Blood samples were collected in both lithium heparin and EDTA tubes prior to surgery (within 48 h of the procedure) and at a 9-month follow-up appointment after surgery. Blood was collected after patients had fasted for at least 6 h.

### Measurement of HbA1c, plasma cholesterol, triglyceride, HDL and LDL concentrations

EDTA-treated whole-blood was used to measure HbA1c by the Laboratory Medicine Service at York Hospital using a boronate affinity chromatography-based method^50^. HbA1c concentrations are expressed as a percentage of total haemoglobin. HbA1c concentrations < 6.0% (< 42 mmol/mol) were considered normoglycemic as per World Health Organisation criteria. Plasma was separated by centrifugation at 2400×*g* for 20 min at 4 °C, snap frozen in liquid nitrogen within 2 h of collection and stored at − 80 °C until analysis. Cholesterol, triglycerides, HDL and LDL cholesterol concentrations were measured in plasma collected in lithium heparin tubes also by the Laboratory Medicine service at York Hospital using standard methods.

### Measurement of plasma fatty acid methyl esters

To measure individual NEFA species present in each sample, NEFAs were converted to fatty acid methyl esters (FAMEs) and analysed by gas chromatography-mass spectrometry (GC- MS) analysis as previously described^51,52^. Plasma samples were spiked with 100 pmol of a heptadecanoate (17:0) internal standard to allow normalisation. NEFAs were extracted using Dole’s method^53^ and converted to FAMEs by incubation with 1.5 ml of methanol, 200 µl of toluene and 300 µl of 8% HCl for 5 h at 45 °C. Samples were evaporated to dryness using nitrogen. The FAME mixtures were then dissolved in 1 ml of water:hexane (50:50 ratio) and the hexane phase was collected and evaporated to dryness in a fume hood. FAMEs were then dissolved in 30 µl dichloromethane and 1–2 µl of the resultant samples were analysed by GC–MS. The instrument used was a GC-6890 N, MS detector-5973 (Agilent Technologies, Santa Clara, CA, USA) with a ZB-5 column (30 m × 25 mm × 25 mm; Phenomenex, Torrance, CA, USA). The temperature program used involved a 70 °C hold for 10 min, followed by a gradient to 220 °C at 5 °C /min, and held at 220 °C for a further 15 min. Mass spectra were acquired from 50–500 amu. FAMEs were identified by comparison of retention times and fragmentation patterns of the samples with various FAME standard mixtures (Supelco, Bellefonte, PA, USA).

## Data Availability

The raw datasets obtained during the current study are available from the corresponding author upon reasonable request.
